# Intraoperative ultrasound in spinal surgery for surgical tailoring and control - A single center case series

**DOI:** 10.1016/j.bas.2025.105891

**Published:** 2025-11-28

**Authors:** Anton Früh, Lukas Depperich, Helen König, Ahmad Almahozi, Joan Alsolivany, Lars Wessels, Peter Vajkoczy, Nils Hecht

**Affiliations:** Department of Neurosurgery, Charité Universitätsmedizin Berlin, Corporate Member of Freie Universität Berlin, Humboldt-Universität zu Berlin and Berlin Institute of Health, Charitéplatz 1, 10117, Berlin, Germany

**Keywords:** Intraoperative ultrasound, Intraoperative imaging, Spine surgery, Spinal cord compression, Non-invasive

## Abstract

**Introduction:**

Intraoperative ultrasound (IOUS) has gained recognition as a valuable imaging modality for enhancing surgical precision in neurosurgical procedures. However, its routine clinical integration in spine surgery remains limited.

**Research question:**

This study aims to elucidate role of intraoperative ultrasound (IOUS) in spinal surgery and to propose the Spinal Cord Pulsatility Index (SCPI) as a novel, ultrasound-based parameter for evaluating spinal cord decompression.

**Material and methods:**

This retrospective single-center case series included all consecutive patients who underwent spinal surgery with IOUS guidance between June 2024 and January 2025. In a subset of patients undergoing posterior decompression, the SCPI – defined as the ratio between the spinal cord pulsation and the corresponding heart rate – was calculated.

**Results:**

Overall, IOUS was performed in 28 patients, and 3 main IOUS applications were determined: (1) anatomic localization, (2) augmentive use, and (3) spinal cord decompression assessment. Importantly, IOUS was fast and technically feasible in all cases, across regions of the spine and regardless of the surgical approach. In a subset of n = 8 cases, we noted a significant SCPI increase at the time-point of final decompression (*p < 0.05).

**Discussion and conclusion:**

IOUS in spinal surgery serves as a simple, safe, cost-effective, and non-invasive imaging modality for real-time localization of intradural and intramedullary pathologies and supplementary neurovascular structures. Based on the principle of communicating fluid dynamics, the spinal cord pulsation index may serve as a novel parameter for indirect assessment of sufficient spinal cord decompression beyond the levels of surgical exposure.

## Highlights

• Intraoperative ultrasound (IOUS) enables fast and safe real-time imaging in spinal surgery
• IOUS supports pathology localization, decompression control, and intraoperative decision-making
• The Spinal Cord Pulsatility Index (SCPI) offers a novel parameter for indirect decompression assessment

## 1 Introduction

Spinal cord compression due to trauma, degenerative diseases, infections, or neoplasms can result in progressive neurological deterioration, manifesting as pain, paresthesia, motor deficits, and loss of autonomic control over bowel and bladder function (Matz et al., 2007). Direct surgical intervention remains the primary treatment for most forms of spinal cord compression, aiming to relieve stress on the neural structures through decompression (Patchell et al., 2005; Badhiwala et al., 2021). However, spine surgery is inherently complex, requiring meticulous anatomical localization and dynamic intraoperative decision-making. The human spine comprises multiple structurally distinct elements that vary in thickness, depth, rigidity, and anatomical configuration (Rauschning, 2017; Ahmed et al., 2018) and pose three principal challenges for intraoperative decision making during spine surgery: First, intraoperative anatomic localization mainly relies on x-ray imaging of the radiographically visible bone structures at the level of interest. However, certain pathologies, such as intradural, extra- and intramedullary lesions cannot be directly localized by radiographic imaging but require visual confirmation after opening of the dura or pia. Second, surgical removal of spinal neoplasms may require supplemental anatomical information, such as the localization of encased vascular structures or the identification of suspected intradural extension in a primarily extradural tumor. Third, to date there is no established tool for non-invasive, intraoperative real-time confirmation of sufficient spinal cord decompression beyond the surgically exposed levels of decompression.

In this context, intraoperative ultrasound (IOUS) has emerged as a valuable adjunct for real time, non-invasive and dynamic imaging in spine surgery (Lurie et al., 2003; Mueller et al., 2006). To date, however, no systematic evaluation has been conducted regarding the overall feasibility of IOUS application across all spinal levels and in a 360-degree surgical approach. Therefore, the aim of the present study was to establish a comprehensive framework for the use of IOUS in pathology localization, intraoperative decision support and evaluation of spinal cord decompression. Furthermore, we introduce a novel parameter based on spinal cord pulsatility, enabling both direct and indirect assessment of decompression. This approach aims to further validate IOUS as a reliable modality for dynamic, non-invasive imaging during spine surgery.

## 2 Materials and methods

### 2.1 Study design and patient population

This single-center retrospective cohort study included patients with spinal pathologies who underwent surgical treatment at our tertiary care center, where intraoperative ultrasound was employed as a supplemental intraoperative imaging modality at the discretion of the primary surgeon. The IOUS examination was performed by the primary surgeon and all consecutive cases that underwent IOUS between June 2024 and January 2025 were included. The study protocol was approved by the local ethics committee (approval number EA4/046/16) and conducted in accordance with the principles of the Declaration of Helsinki. As all treatments adhered to standard clinical procedures at our institution, no additional patient consent was required. However, for cases involving the capture of intraoperative photographs and videos, written informed consent for publication was obtained. All images and videos were fully anonymized to ensure patient confidentiality and prevent any potential identification.

### 2.2 Data collection and analysis

Demographic, radiographic, and clinical data were retrospectively collected and analyzed from patient records in accordance with the Strengthening the Reporting of Observational Studies in Epidemiology guidelines (von Elm et al., 2008).

### 2.3 Intraoperative spine ultrasound

Intraoperative spinal ultrasound was performed using the bkActiv ultrasound system (BK Medical, General Electric Healthcare, Boston, Massachusetts, USA). Two different ultrasound probes, the N13C5 (BK Medical, General Electric Healthcare, Boston, Massachusetts, USA) and N11C5s (BK Medical, General Electric Healthcare, Boston, Massachusetts, USA), were utilized. For intraoperative use, the ultrasound probes were covered with a purpose-designed sterile drape that enabled intraoperative ultrasound imaging by the surgical team without the need for additional gel application. For the purpose of efficient time management, the general setup of the ultrasound device and draping of the probes was performed parallel to the surgery at the beginning of the procedure. To ensure optimal acoustic coupling between the probe and the exposed neural structures during the time point of the measurement, the surgical field was filled with sterile saline solution, creating a water-filled environment.

### 2.4 spinal cord pulsatility index

In cases requiring spinal cord decompression, IOUS was performed in a repetitive fashion to obtain stepwise information on the level of decompression. During this repetitive peri- and post-operative decompression assessment, distinct changes in the oscillatory behavior of the spinal cord were observed, which affected both the pulsation frequency and amplitude of the spinal cord motion within the thecal sac. Since a standardized measure of the pulsation amplitude seemed impractical (e.g. due to different proportions of the spinal cord diameter and the thecal sac diameter across different anatomic levels of the spine), we focused on the pulsation frequency of the spinal cord and hypothesized that this could serve as an indirect measure of spinal cord decompression according to the principle of communicating fluid dynamics (Früh et al., 2023; Benninghaus et al., 2019). For this purpose, we intraoperatively recorded the frequency of peri- and post-decompression spinal cord pulsations within the thecal sac and the patient's corresponding heart rate in a subgroup of n = 8 patients that underwent posterior decompression. A single pulsation was defined as one complete downward and upward movement of the spinal cord and the number of visible spinal cord pulsations was counted during a standardized 30-s interval. Based on these recordings, we calculated the Spinal Cord Pulsatility Index (SCPI) as the ratio between the intrathecal spinal cord pulsations and the corresponding heart rate.

### 2.5 Statistical analysis

Statistical analysis was performed with RStudio. Descriptive summary statistics are presented as median and IQR. The difference between peri- and post-decompression DPI was tested using a Wilcoxon signed-rank testing for dependent samples. The significance level was set at p < 0.05.

## 3 Results

**Fig. 1:**
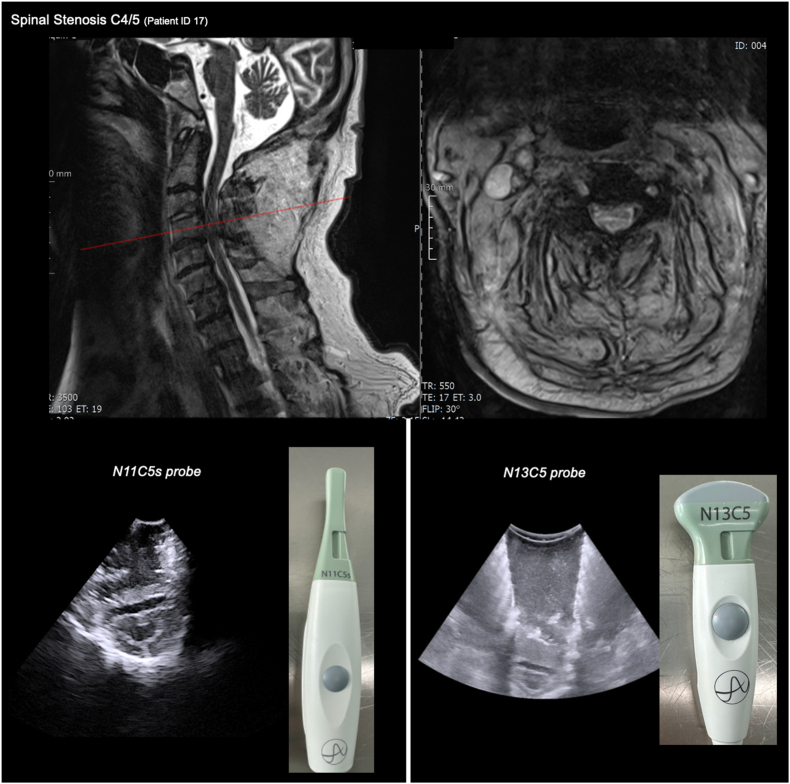
**Utilized ultrasound probes**. Representative case of an 89-year-old patient with a cervical spinal canal stenosis at C4/C5 treated with anterior cervical discectomy and fusion The upper panel shows preoperative T2-weighted MRI images (left: sagittal view; right: axial view). The lower panel illustrates intraoperative ultrasound application: on the left, use of the N11C5S probe; on the right, use of the N13C5 probe.

**Fig. 2:**
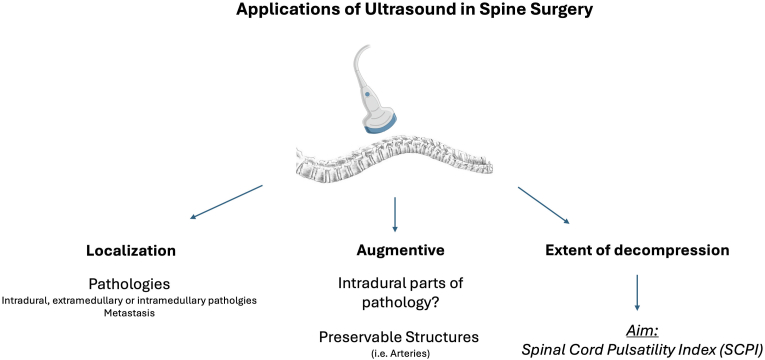
**Applications of Ultrasound in Spine Surgery**. The figure illustrates the three identified clinical applications of intraoperative ultrasound in spine surgery: (1) localization of anatomical structures, (2) augmentative use during surgical procedures, and (3) assessment of spinal cord decompression.

Between June 2024 and January 2025, a total number of 28 patients with a median age of 66 years (IQR 57–74) received IOUS during spine surgery. The female-to-male ratio was 1.5. Individual patient data are presented in Table 1. Intraoperative ultrasound was technically feasible and resulted in a maximum additional surgery time of 3 min. No adverse events were noted. In all cases, the operating surgeons independently opted for the larger N13C5 ultrasound probe due to superior image resolution. A direct comparison of both probes used in the same setting is illustrated in Fig. 1. For the N13C5 probe, the optimal technical ultrasound settings were determined at a frequency of 8 MHz, gain of 12 dB, minimum resolution (36 Hz), thermal index of 0.3, and a mechanical index of 1.06. Overall, 3 distinct types of IOUS application were identified: (1) localization, (2) augmentative use, and (3) spinal cord decompression assessment (Fig. 2). Representative examples of each IOUS application type are presented in the following case illustrations.

**Table 1:** Characteristics of included patients (n = 28).

ID	Age (years)	Diagnosis	Surgical approach	Preoperative Status	Postoperative Status on day 1	LOS (days)	Type of Discharge	Use of intraoperative ultrasound		
								Localization	Augmentive	Decompression
1	84	Fracture T1/T2 with incomplete spinal cord injury	Laminectomy T1/T2 + Posterior fusion C7-T3	Tetraparesis (1/5)	Tetraparesis (3/5)	11	Rehab	no	no	yes
2	66	Spinal stenosis C3/4 + C5/6 with incomplete spinal cord injury	Laminectomy C4-6 + Posterior fusion C3-6	Fine motor impairment, ataxia, paresis upper extremity (2/5)	Unchanged	9	Rehab	no	no	yes
3	59	Fracture C5 with incomplete spinal cord injury	Laminectomy C5 + Posterior fusion C3-6	Paraplegia	Unchanged	16	Rehab	no	no	yes
4	82	Degenerative myelopathy C4/5	Laminectomy C4/5 + Posterior fusion C1-T2	Fine motor impairment, ataxia,	Unchanged	15	Geriatric care	no	no	yes
5	75	Intradural tethered cord T3/4	Hemilaminectomy T4	Bladder dysfunction, claudication	Unchanged	6	Home	yes	yes	no
6	37	Degenerative myelopathy C5-7	Anterior corpectomy C6	Cervicobrachialgia	Improvement of pain	4	Home	no	no	yes
7	65	Fracture C5/6 (Bechterew's Disease)	Laminectomy C5/6 + Posterior fusion C4-T2	Neck Pain	Improvement of pain	8	Home	no	no	yes
8	69	Degenerative myelopathy C3-T2	Laminectomy C3-T2 + Posterior fusion C3-T2	Ataxia	Unchanged	6	Geriatric care	no	no	no
9	72	Cage subsidence after ACDF C4-6	Anterior corpectomy C5 + Posterior fusion C3-T1	Ataxia	Unchanged	5	Home	no	no	no
10	58	Ewing sarcoma T2-5	Decompression T2-6 + Posterior fusion T1-T6	Pain, tumor progression	Improvement of pain	1	Oncology	no	no	yes
11	57	Intramedullary ependymoma T8	Laminectomy T8 + Resection	No deficit	No deficit	4	Home	yes	no	no
12	57	Intra- and extradural Neurinoma C4/5	Anterior resection	Pain, tumor progression	C5 paresis, Horner-syndrome	4	Home	yes	yes	no
13	45	Intramedullary ependymoma T5/6	Laminectomy T5 + Resection	No deficit	No deficit	11	Home	yes	no	yes
14	78	Fracture C6/7 (Bechterew's Disease)	Laminectomy C6/7 + Posterior fusion C5-T2	Neck pain	Improvement of pain	6	Home	no	no	no
15	62	Intradural meningioma C5/6	Hemilaminectomy C5/6 + Resection	Radicular pain	Improvement of pain	8	Home	yes	no	yes
16	89	Degenerative myelopathy C1/2	Laminectomy C1	Dizziness, dysesthesia	Unchanged	4	Home	no	yes	yes
17	75	Degenerative myelopathy C4/5	ACDF C4/5	Ataxia	Improvement of ataxia	3	Home	no	no	yes
18	46	Herniated disc T10/11	Transthoracic sequester removal T10/11	Radicular pain, ataxia	Improvement of pain and ataxia	5	Home	no	no	yes
19	74	Herniated disc T10/11 + Olisthesis L4/5 + Spinal Stenosis T12-L3	TLIF L4/5 + Hemilaminectomy L1-2 + Sequester removal T10/11	Claudication, foot flexor paresis (1/5)	Improvement of claudication	7	Home	yes	no	yes
20	71	Intramedullary sarcoidosis C6/7	Laminectomy C5, Biopsy + Posterior fusion C5/6	Dysesthesia	Unchanged	7	Home	yes	no	yes
21	65	Epidural Hematoma L3-5	Hemilaminectomy L3	Radicular pain	Improvement of pain	13	Home	no	yes	yes
22	35	Intra- and extradural Metastasis L2-4	Decompression L2-4 + Posterior fusion L2-4	Pain, hypesthesia, hip flexor paresis (3/5)	Improvement of pain, hypesthesia and paresis	5	Home	yes	yes	yes
23	67	Recurrent cervicothoracic chordoma	Decompression + Posterior fusion C2-T6	Cervicothoracic pain, dysesthesia	Unchanged	5	Home	no	no	yes
24	76	Metastasis T4	Decompression T5 + Posterior fusion T4-6	Paraplegia	Unchanged	12	Rehab	no	no	yes
25	33	Intradural schwannoma C7-8	Laminectomy C7	Radicular pain	Improvement of pain	4	Home	yes	no	yes
26	47	Intramedullary cavernous malformation	Laminectomy T3	Dysesthesia	Unchanged	8	Home	yes	no	no
27	83	Metastasis C1-C5	Laminectomy + Posterior fusion C1-C6	Radicular and neck pain	Improvement of pain	11	Home	no	no	yes
28	74	Degenerative myelopathy C2-C6, OPLL	Laminectomy + Posterior fusion C2-C5	Ataxia	Improvement of ataxia	6	Rehab	no	no	yes

Abbreviations: LOS = Length of stay, OPLL = Ossification of the Posterior Longitudinal Ligament, ACDF = Anterior Cervical Discectomy and Fusion, TLIF = Transforaminal Lumbar Interbody Fusion, (paresis according to the MRC scale).

### 3.1 Application type 1 – pathology localization (n = 10)

**Fig. 3:**
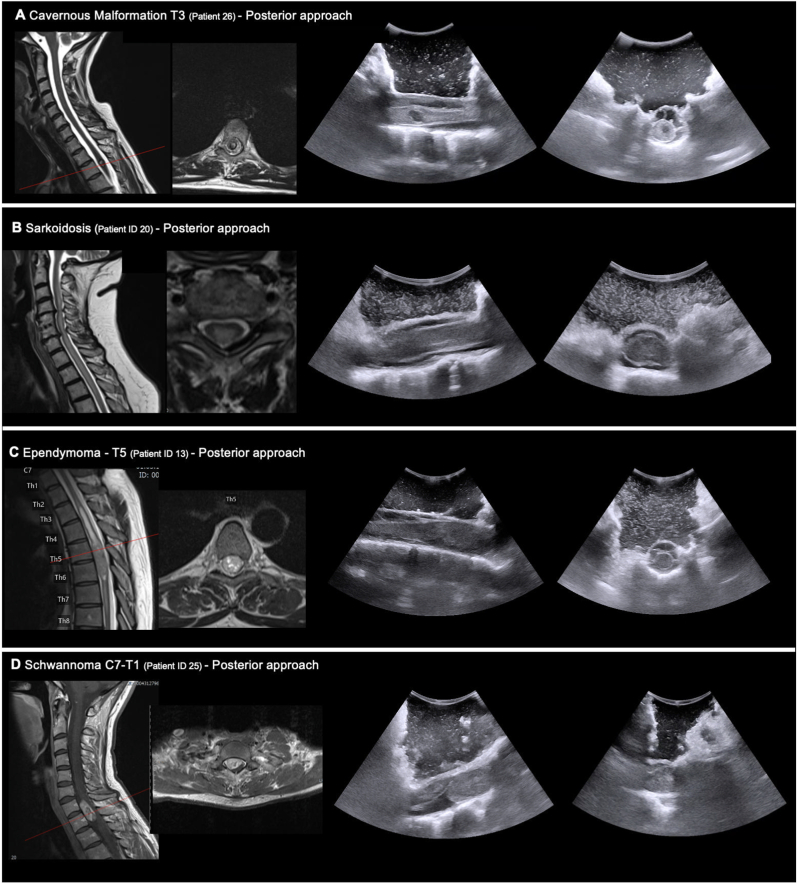
**Application of ultrasound for Localization.** Illustration of four representative cases in which intraoperative ultrasound was utilized for anatomical localization. All patients underwent posterior surgical approaches. In each case, the left panels show preoperative sagittal and axial MRI scans, and the corresponding right panels show intraoperative sagittal and axial ultrasound images. **A**. 47-year-old male patient with an intramedullary cavernous malformation at T3. **B**. 71-year-old female patient with spinal sarcoidosis. **C**. 45-year-old male patient with an intramedullary ependymoma. **D**. 33-year-old female patient with an intradural schwannoma at C7.

In intradural extramedullary lesions, IOUS was helpful for confirming the correct level of exposure and permitted individual tailoring the lamina removal to permit optimal surgical exposure beyond the cranial and caudal tumor extent, which facilitated gentle tumor removal. For intradural intramedullary lesions, Fig. 3 shows case examples of a cavernous malformation, sarcoidosis, and ependymoma that were not clearly visible at the surface of the spinal cord. Importantly, IOUS was used before and after opening of the dura, so that IOUS was valuable for confirming the correct level of surgical exposure but also for identifying the site for opening of the pia and myelotomy in order to minimize the risk of surgical morbidity.

### 3.2 Application type 2 – augmentive use (n = 5)

**Fig. 4:**
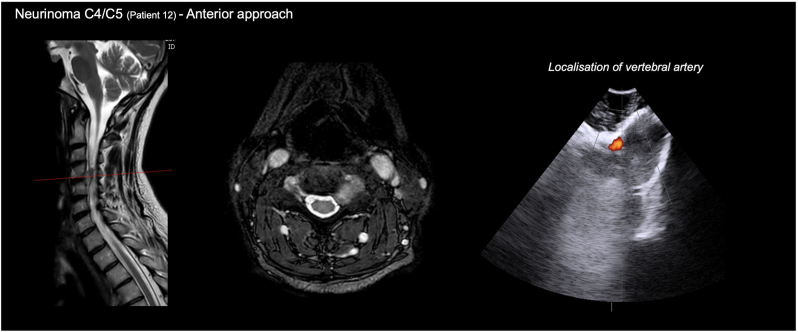
**Augmentive Application of intraoperative Ultrasound**. Representative case of a 57-year-old female patient with a C4/C5 neurinoma treated via an anterior approach. The left and middle panels show preoperative T2-weighted MRI in sagittal and axial views. The right panel displays the intraoperative ultrasound image, which enabled visualization of the vertebral artery to ensure its preservation during tumor resection.

Fig. 4 illustrates the augmentive use of IOUS during resection of a combined intra- and extradural cervical schwannoma at the C5 level through an anterolateral approach. In this patient, the tumor had encased the left vertebral artery. After exposure and visual identification of the anterior tumor surface, IOUS and doppler sonography was used for dynamic real-time visualization of the tumor and the encased vertebral artery. This markedly facilitated anatomic orientation and permitted a safe and targeted dissection of the vertebral artery, which required resection of the C4 and C5 transverse processes and circumferential mobilization of the vertebral artery through the anterolateral corridor to allow complete tumor resection. Importantly, this augmentive use highlights the utility of IOUS in spine procedures where radiographic imaging or the use of spinal navigation is not technically feasible or helpful.

### 3.3 Application type 3 – spinal cord decompression assessment (n = 21)

**Fig. 5:**
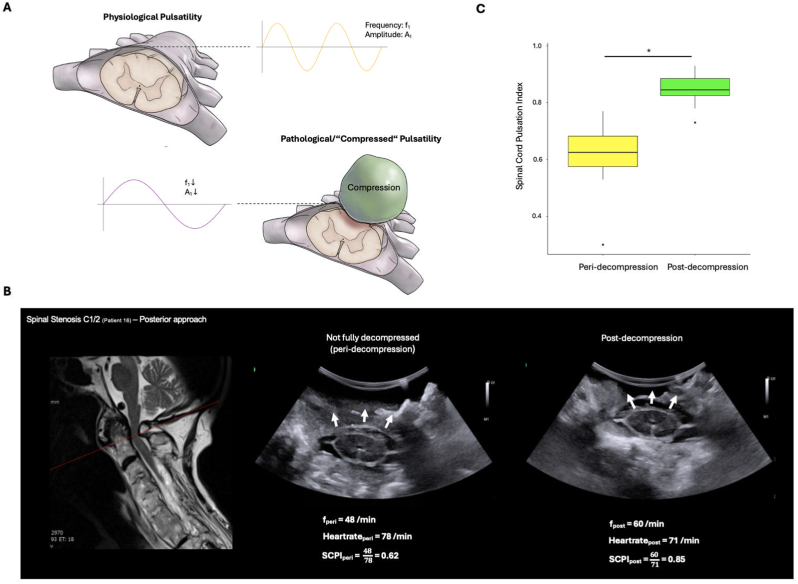
Application of ultrasound for assessment of the extent of decompression. **A**. Schematic illustration of the hypothesis underlying the development of the spinal cord pulsatility index (SCPI). The upper panel depicts normal spinal cord pulsation, characterized by physiological frequency and amplitude. The lower panel illustrates spinal cord decompression, which may alter both the frequency and amplitude of dural pulsations. **B**. Representative case of an 89-year-old female patient with a C1/C2 spinal canal stenosis. The left panel shows the preoperative T2-weighted MRI. The adjacent images demonstrate intraoperative assessment of SCPI, including measurements peri-decompression and post-decompression. **C**. Box plot of SCPI values peri- and post-decompression (n = 8), showing a statistically significant difference (p < 0.01).

Fig. 5B illustrates the use of IOUS for assessing decompression in a patient with degenerative cervical myelopathy who underwent posterior decompression. In this setting, IOUS permitted dynamic 360-degree real-time visualization of the spinal cord pulsation and the surrounding cerebrospinal fluid, which served as a direct sign for sufficient 360-degree decompression in the imaged area.

### 3.4 Spinal cord pulsatility (n = 8)

Despite the possibility of direct decompression assessment, IOUS does not permit direct visual assessment of spinal cord decompression beyond the surgically exposed levels. To address this issue, we defined the Spinal Cord Pulsatility Index (SCPI) as a parameter for indirect spinal cord decompression assessment based on communicating intrathecal fluid dynamics (Fig. 5A). Representative SCPI calculations are shown in Fig. 5B. Overall, peri- and post-decompression assessment was performed in n = 8 patients with a significant SCPI increase after final decompression (p < 0.01 for peri-vs. post-decompression SCPI; Fig. 5C). Further examples of IOUS are shown in **Video 1.**

## 4 Discussion

The principal finding of this study is that IOUS during spine surgery can serve as a simple, safe, cost-effective and non-invasive imaging modality for pathology localization, anatomy assessment and decompression control during spine surgery across all regions of the spine. In addition, the spinal cord pulsatility index may serve as an indirect parameter to determine sufficient spinal cord decompression beyond the surgically exposed levels.

### 4.1 Intraoperative imaging

Intraoperative imaging has emerged as a valuable modality to improve quality control and safety aspects for patients undergoing spine surgery (Kendlbacher et al., 2022; Tkatschenko et al., 2020; Noriega et al., 2017), but routine utilization of magnetic resonance imaging (MRI), computed tomography (CT) or cone-beam CT for the purpose of dynamic real-time pathology localization, anatomy assessment and decompression control is hampered due to technical, financial and logistical reasons. Against this background, ultrasound represents a mobile, non-invasive, cost-effective imaging modality that can provide continuous and dynamic intraoperative feedback in real-time (Gooding et al., 1984). Importantly, IOUS does not expose patients and the OR-team to radiation. This permits unlimited image acquisition, which we experienced helpful during various types of IOUS application that we described and in particularly for intradural pathology localization, vascular doppler imaging and spinal cord decompression assessment. Together, this provides new opportunities for soft-tissue imaging in spine surgery and holds potential to decrease the overall radiation exposure for the patients as well as the OR-team (Wang et al., 2011; Patel et al., 2022). Another advantage of IOUS that we observed with the larger, high-resolution ultrasound probe is high spatial and temporal resolution, which facilitated precise localization of small intramedullary pathologies, for example in a patient who underwent spinal cord biopsy and was diagnosed with sarcoidosis (Fig. 3B). This high spatial-temporal resolution was also helpful for assessment of spinal cord decompression, since visually adequate circumferential decompression is crucial for clinical success (Alaqeel et al., 2015). In addition, the high temporal resolution permitted counting of spinal cord pulsations in real-time for calculation of the SCPI. While MRI provides excellent spatial resolution and anatomical detail, its intraoperative application remains limited by a stationary design, high cost, restricted availability and the personnel requirements. Currently, intraoperative MRI is primarily used in cranial tumor surgery, where clear evidence for improved clinical outcome is still lacking (de Los Reyes-Nabhan et al., 2025). For the purpose of direct intraoperative real-time assessment, MRI remains further limited by time-consumption and static image acquisition, which together with the logistical and personnel challenges limits the 24/7 availability and simple utilization of the technology compared to IOUS. Although CT and cone-beam CT may enable more rapid intraoperative image assessment, both are associated with radiation exposure and mainly revolve around verification of bony decompression and implant positioning with very limited soft-tissue contrast (Haemmerli et al., 2023; Krishnan et al., 2025). Furthermore, MRI, CT and cone-beam CT all lack continuous and dynamic real-time imaging properties, required for in situ recording of dynamic oscillations of the spinal cord within cerebrospinal fluid. On the other hand, MRI, CT and cone-beam CT have the clear advantage of less operator dependency and reliably provide highly standardized images in excellent quality, whereas IOUS requires greater experience for reliable image acquisition and interpretation. Despite these benefits and limitations, however, each of the above-mentioned intraoperative imaging technologies has yet to prove their clear benefit for patient outcomes in prospective clinical trials.

From an economical perspective the unlimited repeatability, mobility and seamless workflow integration of IOUS appeared highly efficient. Apart from the device and disposable sterile drapes, no additional resources were required. The mobility of the device permitted parallel use in different operating rooms. Further, ultrasound imaging was performed by the surgical team without requiring any specific qualification, compared to other intraoperative imaging technologies frequently used in spine surgery that are subject to radiation protection regulations (Kendlbacher et al., 2022). Also, IOUS utilization was fast and did not lead to a relevant prolongation of the OR time, which indicates a seamless workflow integration. The ultrasound system that we used also permitted direct integration into picture archiving and communication systems, thereby ensuring efficient documentation and postoperative review. Nevertheless, we recommend routine utilization of IOUS in every spine surgery case to continuously optimize workflow, train for proficiency and improve familiarity with handling and image interpretation.

### 4.2 Spinal cord pulsatility index (SCPI)

Although IOUS enabled direct assessment of spinal cord decompression, ultrasound cannot penetrate bone. Therefore, direct decompression assessment beyond areas of the exposed thecal sac is naturally limited. To address this issue, we explored the feasibility of indirect decompression assessment based on the principle of communicating intrathecal fluid dynamics. Specifically, we hypothesized that heart rate oscillations should be directly transmitted along a freely communicating (decompressed) intrathecal spinal cord floating within CSD, because physiological cerebrospinal fluid dynamics are primarily governed by pulsatile perfusion pressure, which is driven by arterial blood pressure (Früh et al., 2023). Essentially, this pressure shift causes cerebrospinal fluid to move bidirectionally between the more compliant spinal compartment and the intracranial compartment during systole and diastole (Benninghaus et al., 2019). Depending on the degree of CSF flow obstruction, for example by spinal cord compression, the frequency and amplitude of intrathecal spinal cord oscillations would be subject to measurable alterations. This hypothesis falls in line with a previous observation that the oscillatory behavior of arterial pressure correlates with lumbar pressure and can provide relevant information for intracranial pressure monitoring (Früh et al., 2023). However, due to the susceptibility of the pulsation amplitude to multiple confounding factors, such as the proportion of the spinal cord diameter to the thecal sac diameter, positive end-expiratory pressure, and variations in systolic/diastolic blood pressure, we decided to focus on the pulsation frequency instead of the amplitude to obtain a more robust and generalizable imaging parameter. Interestingly, our present findings showed a significant increase of the ratio between spinal cord pulsation and the corresponding heart rate at the end compared to the beginning of the decompression, which falls in line with our hypothesis and suggests an improvement in intrathecal CSF flow along the spinal cord as an indirect sign of sufficient spinal cord decompression beyond the surgically exposed levels. Of course, this hypothesis requires further testing in a larger cohort through systematic IOUS in pre-specified pathologies during standardized approaches together with postoperative imaging and outcome assessment, but nevertheless, we believe that our SCPI concept could facilitate the establishment of threshold values indicative of sufficient decompression, thereby providing a potential objective criterion for intraoperative surgical decision-making.

### 4.3 Limitations and future studies

This study is subject to several limitations: First, the small sample size and single-center, university-based setting may limit the generalizability of our findings to other institutions, particularly those with different patient populations and equipment availability. Second, the retrospective design introduces inherent biases, including selection bias and missing data, which could impact the robustness of our conclusions. Third, IOUS is highly operator-dependent, with image acquisition and interpretation varying based on the surgeon's experience. This variability may affect reproducibility, especially in centers with limited exposure to IOUS. Standardized training protocols and interobserver reliability assessments should be explored in future studies to address this limitation. Forth, while the proposed SCPI offers a novel approach to assessing spinal decompression, its clinical significance remains uncertain due to the lack of correlation with postoperative imaging or validated functional outcome measures, such as the modified Japanese Orthopedic Association score. Further prospective studies are needed to establish its predictive value and determine potential threshold values for adequate decompression. Another challenge lies in the general measurement of the frequency and amplitude of spinal cord pulsatility, which is influenced by multiple physiological and technical confounders. Variations in arterial blood pressure, positive end-expiratory pressure, cerebrospinal fluid dynamics, and patient positioning may all impact intrathecal spinal cord motion, potentially limiting the reliability of SCPI as an independent marker of decompression. Lastly, while we opted to focus on spinal cord pulsation frequency rather than amplitude for reasons outlined above, amplitude changes may still hold clinical significance and warrant further investigation. Therefore, prospective studies with larger cohorts and multicenter participation are warranted to strengthen the conclusions and provide greater statistical power. Standardized training protocols for surgeons and interobserver reliability assessments will be essential to improve reproducibility. A key objective of upcoming studies should be the systematic identification and quantification of physiological and technical confounders influencing SCPI measurements. Consequently, future protocols should include end-expiratory, cardiac-gated acquisitions during hemodynamically stable periods, standardized ventilation and positioning, and synchronized recording of blood pressure, Positive End Expiratory Pressure, and end-tidal CO_2_. Normalization of SCPI values to pulse pressure or, blood pressure combined with predefined mixed-effects models and sensitivity analyses could enable more accurate adjustment for these confounding factors. Operator dependency should be further mitigated by structured training, quality checklists, and interobserver reliability assessments, ideally supported by (semi)-automated measurement workflows. Most importantly, future studies should integrate longitudinal follow-up data on patient recovery, functional improvement, and quality of life after surgery to comprehensively assess the clinical impact of IOUS-guided decompression and determine whether intraoperative SCPI changes correlate with neurological and functional outcomes.

## 5 Conclusion

Ultrasound represents a simple, safe and fast tool in spinal surgery, providing highly accurate real-time imaging with the potential to enhance surgical precision and assist in intraoperative decision-making. Therefore, integration of IOUS holds the potential to serve as a means quality control and optimization of clinical outcomes in spine surgery.

## Statement and declarations

The authors have nothing to declare and there is no potential conflict of interest.

## Conflict of interest

We confirm that the manuscript complies with all instructions to authors, further we confirm that the authorship requirements have been met and the final manuscript was approved by all authors. We confirm that this manuscript has not been published elsewhere and is not under consideration by another journal. Lead ethics approval was approved and the study was performed according to the Declaration of Helsinki. There is no conflict of interest of any of the authors. The study received no funding. Dr. Früh funded by the BIH Charité Junior Digital Clinician Scientist Program of the Charité – Universitätsmedizin Berlin, and the Berlin Institute of Health at Charité (BIH).

## Appendix A Supplementary data

Video 1. The video demonstrates four representative clinical cases in which intraoperative ultrasound was applied. In each case, ultrasound was used to evaluate the pulsatility.Multimedia component
